# The combination of chidamide with the CHOEP regimen in previously untreated patients with peripheral T-cell lymphoma: a prospective, multicenter, single arm, phase 1b/2 study

**DOI:** 10.20892/j.issn.2095-3941.2020.0413

**Published:** 2021-08-15

**Authors:** Wei Zhang, Liping Su, Lihong Liu, Yuhuan Gao, Quanshun Wang, Hang Su, Yuhuan Song, Huilai Zhang, Jing Shen, Hongmei Jing, Shuye Wang, Xinan Cen, Hui Liu, Aichun Liu, Zengjun Li, Jianmin Luo, Jianxia He, Jingwen Wang, O. A. O’Connor, Daobin Zhou

**Affiliations:** 1 Department of Hematology, Peking Union Medical College Hospital, Beijing 100730, China; 2 Department of Hematology, Shanxi Provincial Cancer Hospital, Taiyuan 030013, China; 3 Department of Hematology, Fourth Hospital of Hebei Medical University, Shijiazhuang 050011, China; 4 Department of Hematology, Chinese PLA General Hospital, Beijing 100039, China; 5 Department of Lymphoma, the 307 Hospital of PLA, Beijing 100071, China; 6 Department of Lymphoma, Peking University Cancer Hospital and Institute, Beijing 100142, China; 7 Department of Lymphoma, Tianjin Medical University Cancer Institute and Hospital, National Clinical Research Center for Cancer, Key Laboratory of Cancer Prevention and Therapy, Tianjin, Tianjin’s Clinical Research Center for Cancer, Tianjin 300060, China; 8 Department of Hematology, Beijing Friendship Hospital, Beijing 100050, China; 9 Department of Hematology, Peking University Third Hospital, Beijing 100191, China; 10 Department of Hematology, the First Affiliated Hospital of Harbin Medical University, Harbin 150081, China; 11 Department of Hematology, Peking University First Hospital, Beijing 100034, China; 12 Department of Hematology, Beijing Hospital, Beijing 100730, China; 13 Department of Lymphoma, Harbin Medical University Cancer Hospital, Harbin 150081, China; 14 Lymphoma Diagnosis and Treatment Center, Institute of Hematology and Blood Diseases Hospital, Tianjin 300052, China; 15 Department of Hematology, Second Hospital of Hebei Medical University, Shijiazhuang 050000, China; 16 Department of Hematology, Shanxi Provincial People’s Hospital, Taiyuan 030012, China; 17 Department of Hematology, Beijing Tongren Hospital, Beijing 100730, China; 18 Columbia University Medical Center, New York 10032-3784, NY, USA

**Keywords:** Peripheral T-cell lymphoma, chidamide, histone deacetylase inhibitor, epigenetic

## Abstract

**Objective:**

To assess the efficacy and safety of the novel histone deacetylase inhibitor, chidamide, in combination with cyclophosphamide, doxorubicin, vincristine, etoposide, and prednisone (Chi-CHOEP) for untreated peripheral T-cell lymphoma (PTCL).

**Methods:**

A prospective, multicenter, single arm, phase 1b/2 study was conducted. A total of 128 patients with untreated PTCL (18–70 years of age) were enrolled between March 2016 and November 2019, and treated with up to 6 cycles with the Chi-CHOEP regimen. In the phase 1b study, 3 dose levels of chidamide were evaluated and the primary endpoint was determination of the maximum-tolerated dose and recommended phase 2 dose (RP2D). The primary endpoint of the phase 2 study was 2-year progression-free survival (PFS).

**Results:**

Fifteen patients were enrolled in the phase 1b study and the RP2D for chidamide was determined to be 20 mg, twice a week. A total of 113 patients were treated at the RP2D in the phase 2 study, and the overall response rate was 60.2%, with a complete response rate of 40.7%. At a median follow-up of 36 months, the median PFS was 10.7 months, with 1-, 2-, and 3-year PFS rates of 49.9%, 38.0%, and 32.8%, respectively. The Chi-CHOEP regimen was well-tolerated, with grade 3/4 neutropenia occurring in approximately two-thirds of the patients. No unexpected adverse events (AEs) were reported and the observed AEs were manageable.

**Conclusions:**

This large cohort phase 1b/2 study showed that Chi-CHOEP was well-tolerated with modest efficacy in previously untreated PTCL patients.

## Introduction

Peripheral T-cell lymphomas (PTCLs) constitute a group of heterogeneous lymphomas, mainly including PTCL-not otherwise specified (PTCL-NOS), angioimmunoblastic T-cell lymphoma (AITL), anaplastic large cell lymphoma (ALCL), and other rare entities. PTCLs account for 10%–15% of all non-Hodgkin lymphomas (NHLs) worldwide, being significantly higher in China (23.3%–30.2%)^1,2^. No standard treatments have been established and commonly used (cyclophosphamide, doxorubicin, vincristine, and prednisone (CHOP) or etoposide with CHOP (CHOEP) regimens result in poor outcomes, except for anaplastic lymphoma kinase (ALK)-positive ALCL^3^. Upfront autologous stem cell transplantation (ASCT) has been recommended to improve the prognosis of PTCL^4^. Frequent epigenetic dysregulation has been documented in patients with PTCL, which makes histone deacetylase (HDAC) a potential target^5^. Several HDAC inhibitors have been approved for PTCL in a relapsed/refractory setting, such as belinostat^6^, romidepsin^7^, and chidamide^8^.

Chidamide, a novel benzamide class of HDAC inhibitor, has demonstrated broad spectrum activity against various neoplasms^9^. In a pivotal phase 2 trial with refractory or relapsed PTCL, chidamide produced an overall response rate (ORR) and complete response (CR) rate of 28% and 14%, respectively, leading to approval by the National Medical Products Administration (China) in 2014 for treatment of refractory or relapsed PTCL^8^. Whether first-line use of HADC inhibitors in combination with the CHOEP regimen will further improve the prognosis in PTCL is unknown. Thus, we conducted a prospective, multicenter, phase 1b/2 study to evaluate the efficacy and safety of combination chidamide and CHOEP (Chi-CHOEP) in previously untreated patients with PTCL, hoping to establish a more effective regimen for this aggressive lymphoma.

## Materials and methods

### Study design and participants

A prospective, multicenter, single arm, phase 1b/2 study was conducted at 17 sites across China to evaluate the safety and efficacy of Chi-CHOEP in previously untreated patients with PTCL (ClinicalTrials.gov Identifier: NCT02987244). This study was carried out in accordance with the principles of good clinical practice and the Declaration of Helsinki. The protocol, informed consent forms, and other relevant study documentation were approved by the Ethics Committee of each center. All patients provided written informed consent before study entry.

**Figure 1:**
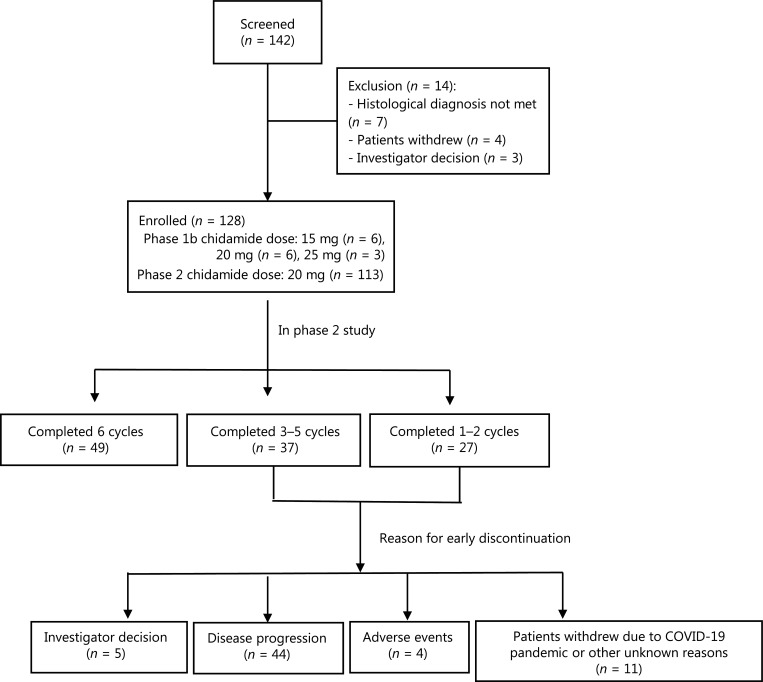
Flow chart of this phase 1b/2 study.

A total of 142 patients (18–70 years of age) were screened and 128 were enrolled between March 1, 2016 and November 30, 2019 (**Figure 1**). The diagnosis of PTCL was confirmed according to the 2016 WHO classification^10^, and ALCL (ALK+), NK/T-cell lymphoma, and primary cutaneous lymphoma were excluded.

### Treatment protocol

The treatment protocol involved induction therapy with up to 6 cycles of the Chi-CHOEP regimen as follows: CHOEP [intravenous cyclophosphamide (750 mg/m^2^), doxorubicin (50 mg/m^2^), or epirubicin (70 mg/m^2^), and vincristine (1.4 mg/m^2^) or vindesine (4 mg on day 1), etoposide (100 mg on days 1–3), and oral prednisone (60 mg/m^2^ on days 1–5), repeated every 21 days] in combination with oral escalating doses of chidamide twice a week. Patients were restaged by computed tomography (CT) or positron emission tomography-CT after the first 3 cycles according to the revised response criteria for NHL. Patients with stable disease (SD) or progressive disease (PD) discontinued study treatment and went on to receive salvage therapy at the discretion of the treating physician. Patients with a complete response or partial response received 3 additional cycles to complete the planned 6 cycles. The selection of subsequent therapy was left to the discretion of the treating physician (either ASCT consolidation or chidamide maintenance).

### Study endpoints

This study consisted of dose-escalation (phase 1b) and expansion (phase 2) phases. In phase 1b, 3 dose levels of chidamide (15, 20, and 25 mg twice a week) were evaluated at a traditional 3 + 3 dose-escalation design. Dose-limited toxicity (DLT) was defined in the first 2 cycles and predefined DLTs included non-hematological toxicity of any grade ≥ 3, grade 4 agranulocytosis with granulocyte colony-stimulating factor support, or grade 4 thrombocytopenia lasting > 7 days. The primary endpoint of the phase 2 study was 2-year progression-free survival (PFS), and secondary endpoints included overall response rate (ORR), CR, 2-year overall survival (OS), and adverse events (AEs). PFS was defined as the time from treatment initiation until disease progression, death from any cause, or last follow-up, whichever came first.

### Statistical analysis

All statistical analyses were performed with SPSS statistical software for Windows, version 24.0 (SPSS, Chicago, IL, USA). The ORR and CR rates were compared with a cross-tabulation χ^2^ test. The PFS and OS were estimated using the Kaplan-Meier method and log-rank test. Patients who were lost to follow-up were censored at their last follow-up date. The threshold for statistical significance was set at a *P* < 0.05 with two-tailed tests. All analyses were completed on an intention-to-treat (ITT) basis.

## Results

### Patient characteristics

As is shown in **Figure 1**, a total of 142 patients were screened at 17 sites across China. Fourteen patients were excluded, mainly due to unmatched diagnoses. The baseline characteristics are shown in **Table 1**. The enrolled patients mainly had advanced disease [stages III–IV (93.3% in phase 1b and 78.8% in phase 2)] and medium-to-high risk disease [International Prognostic Index score: 2–5 (100% in phase 1b and 69.9% in phase 2)]. The most common subtypes in phase 2 included PTCL-NOS (42.5%), AITL (36.3%), and ALK-negative ALCL [ALK^-^ALCL (15.0%)].

**Table 1:** Patient baseline characteristics

Characteristics	Phase 1b (*n* = 15), *n* (%)	Phase 2 (*n* = 113), *n* (%)
Age, years		
Median	54	54
Range	24–69	20–70
> 60	4 (26.7)	33 (29.2)
Male	9 (60.0)	72 (63.7)
Ann Arbor staging		
Stage I	0 (0)	5 (4.4)
Stage II	1 (6.7)	19 (16.8)
Stage III	4 (26.7)	32 (28.3)
Stage IV	10 (66.7)	57 (50.4)
B symptoms	10 (66.7)	66 (58.4)
IPI		
0–1	0 (0)	34 (30.1)
2	6 (40.0)	33 (29.2)
3	4 (26.7)	24 (21.2)
4–5	5 (33.3)	22 (19.5)
Pathology subtypes		
AITL	9 (60.0)	41 (36.3)
PTCL-NOS	1 (7.0)	48 (42.5)
ALCL, ALK-	4 (26.0)	17 (15.0)
EATL	1 (7.0)	7 (6.2)

AITL, angioimmunoblastic T cell lymphoma; PTCL-NOS, peripheral T cell lymphoma-not otherwise specified; ALCL, anaplastic large cell lymphoma; EATL, enteropathy-associated T-cell lymphoma; IPI, international prognostic index.

### Recommended dose of chidamide

As is shown in **Table 2**, 15 patients were enrolled in phase 1b. At the 15 mg dose, 1 DLT of grade 3 pneumonia occurred, and 1 DLT of grade 4 neutropenia developed at the 20 mg dose. At the 25 mg dose, 2 DLTs of grade 4 neutropenia occurred, thus 20 mg was determined to be the maximum tolerated dose (MTD) and recommended phase 2 dose (RP2D).

**Table 2:** DLTs in the phase 1b study

Chidamide dose	Patients treated (*n*)	DLT (*n*)	Toxicity	Best response
15 mg biw	6	1	Grade 3 pulmonary infection	2CR, 1PR
20 mg biw^1^	6	1	Grade 4 neutropenia with G-CSF support	2CR
25 mg biw	3	2	Grade 4 neutropenia with G-CSF support	2PR

DLT, dose limited toxicity; CR, complete response; PR, partial response; G-CSF, granulocyte colony-stimulating factor.

^1^Maximum tolerated dose (MTD) and Recommended Phase 2 Dose (RP2D).

### Treatment efficacy

In the phase 2 study, 113 patients were enrolled, and a median of 5 cycles (range: 1–6) of Chi-CHOEP was administered. The reasons for premature discontinuation mainly included disease progression [*n* = 44 (38.9%)], AEs [*n* = 4 (3.5%)], and withdrawal of informed consent [*n* = 11 (9.7%)] due to the COVID-19 pandemic and other unknown reasons. As shown in **Table 3**, the response could be assessed in 109 patients (96.5%). The ORR was 60.2% in the ITT cohort (*n* = 113), with a CR rate of 40.7%. Patients with low risk disease had a significantly higher ORR and CR than those with high risk disease (*P* < 0.05). Based on the pathology subtypes, patients with ALK^-^ALCL had the best ORR, followed by AITL and PTCL-NOS; the worst ORR occurred in patients with EATL (76.5% *vs.* 65.9% *vs.* 54.2% *vs.* 28.6%). No significant difference in response rate was found between patients who received doxorubicin *vs.* epirubicin (P > 0.05).

**Table 3:** Efficacy assessment of the phase 2 study

Characteristics	ORR (%)	CR (%)	SD (%)	PD (%)	Not evaluable (%)*
Phase 2 (*n* = 113)	68 (60.2)	46 (40.7)	11 (9.7)	30 (26.5)	4 (3.5)
IPI					
0–1 (*n* = 34)	23 (67.6)	18 (52.9)	3 (8.8)	7 (20.6)	1 (2.9)
2 (*n* = 33)	20 (60.6)	13 (39.4)	6 (18.2)	7 (21.2)	0 (0)
3 (*n* = 24)	14 (58.3)	7 (29.2)	1 (4.2)	8 (33.3)	1 (4.2)
4–5 (*n* = 22)	11 (50.0)	8 (36.4)	1 (4.5)	9 (40.9)	2 (9.1)
Pathology subtypes					
AITL (*n* = 41)	27 (65.9)	17 (41.5)	4 (9.8)	8 (19.5)	2 (4.9)
PTCL-NOS (*n* = 48)	26 (54.2)	20 (41.7)	5 (10.4)	16 (33.3)	1 (2.1)
ALCL (ALK-) (*n* = 17)	13 (76.5)	8 (47.1)	2 (2.8)	2 (2.8)	0 (0)
EATL (*n* = 7)	2 (28.6)	1 (14.3)	0 (0)	4 (57.1)	1 (14.3)

*Patients without efficacy assessments due to premature discontinuation by AEs.

ORR, overall response rate; CR, complete response; SD, stable disease; PD, progressive disease; IPI, international prognostic index; AITL, angioimmunoblastic T cell lymphoma; PTCL-NOS, peripheral T cell lymphoma-not otherwise specified; ALCL, anaplastic large cell lymphoma; EATL, enteropathy-associated T-cell lymphoma; AE, adverse event.

### Safety profiles

Overall, this regimen was well-tolerated, with grade 3/4 neutropenia occurring in approximately two-thirds of the patients. No unexpected AEs were reported and the observed AEs were manageable, and consistent with what was expected with the CHOEP regimen. **Table 4** summarizes the most frequently encountered hematological and non-hematological AEs.

**Table 4:** Adverse events of the phase 1b/2 study

AE	Phase 1b (*n* = 15)				Phase 2 (*n* = 113)			
	Any grade		Grade ≥ 3		Any grade		Grade ≥ 3	
	*n*	%	*n*	%	*n*	%	*n*	%
Leucopenia	14	93.3	9	60.0	93	82.3	78	69.0
Anemia	11	73.3	4	26.7	93	82.3	42	37.2
Neutropenia	11	73.3	10	66.7	86	76.1	78	69.0
Thrombocytopenia	8	53.3	5	33.3	58	51.3	35	31.0
Nausea/vomiting	6	40.0			72	63.7	5	4.4
Fever	4	26.7	2	13.3	42	37.2	12	10.6
Hypokalemia	4	26.7			34	30.1	17	15.0
Hypocalcaemia	8	53.3	1	6.7	25	22.1	3	2.7
Hypoalbuminemia	4	26.7			32	28.3	2	1.8
Upper respiratory tract infection	4	26.7	1	6.7	20	17.7	3	2.7
Pulmonary infection	1	6.7	1	6.7	15	13.3	7	6.2
Fatigue	3	20.0	1	6.7	12	10.6		
Hyponatremia	2	13.3	2	13.3	12	10.6	12	10.6
Hyperglycemia	2	13.3			12	10.6	7	6.2
ALT/AST elevation	2	13.3			8	7.1	3	2.7
BUN elevation	1	6.7			8	7.1	3	2.7
Skin rash	1	6.7			7	6.2		
Lower limb edema					7	6.2	2	1.8

Plasma Epstein-Barr virus (EBV) DNA copy number was examined in 55 of the 113 treated patients at the study entry. Baseline EBV-DNA was detectable in 5 patients and undetectable in 50 patients. No EBV reactivation throughout the duration of treatment was detected in any of the 5 patients with detectable baseline EBV-DNA. Among the 50 patients with undetectable EBV-DNA at baseline, only 1 patient displayed an increase in the EBV-DNA copy number. This patient died due to disease progression. Hepatitis B virus (HBV) DNA was examined in 65 of the 113 enrolled patients at study entry, and no HBV reactivation was observed throughout treatment.

### Survival outcomes analysis

**Figure 2:**
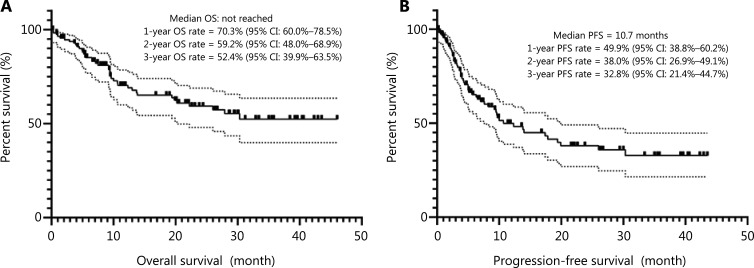
Kaplan-Meier survival curves for the phase 2 cohort. (A) Overall survival. (B) Progression-free survival.

**Figure 3:**
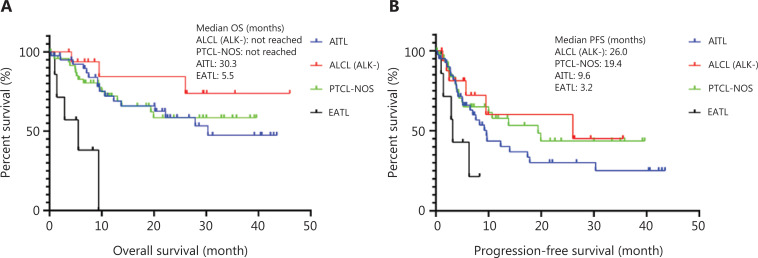
Subgroup analysis of survival outcomes by pathology subtypes. (A) Overall survival. (B) Progression-free survival. AITL, angioimmunoblastic T cell lymphoma; PTCL-NOS, peripheral T cell lymphoma-not otherwise specified; ALCL, anaplastic large cell lymphoma; EATL, enteropathy-associated T-cell lymphoma.

At a median follow-up of 36 months (range: 7.3–51.9 months), the median PFS was 10.7 months, with 1-, 2-, and 3-year PFS rates of 49.9%, 38.0%, and 32.8%, respectively (**Figure 2A**). In the phase 2 cohort, 55 patients had disease progression or relapse at a median time of 4.2 months (0.3–30.3), with 64% patients suffering from disease progression within 6 months since initiation of Chi-CHOEP treatment. For patients who received CR after Chi-CHOEP treatment, only 24% (11 of 46) had relapsed diseases at a median time of 9.7 months (4.2–19.9 months). As shown in **Figure 3A**, patients with ALK^-^ALCL had the longest median PFS (26.0 months), followed by PTCL-NOS (19.4 months), AITL (9.6 months), and EATL (3.2 months; *P* < 0.05). The median OS was not reached for the entire cohort, with 1-, 2-, and 3-year OS rates of 70.3%, 59.2%, and 52.4%, respectively (**Figure 2B**). Patients with AITL and EATL had significantly inferior outcomes compared with ALK^-^ALCL and PTCL-NOS patients (*P* < 0.05; **Figure 3B**).

## Discussion

This large cohort phase 1b/2 study showed for the first time that chidamide added to CHOEP was well-tolerated with modest efficacy in previously untreated PTCL patients. The RP2D of chidamide was determined to be 20 mg twice a week when combined with CHOEP, and this dosage should be used in future clinical practice.

No standard first-line treatment has been defined for patients with PTCL except for the ALK positive ALCL patients. According to the data reported by the German High-Grade Non-Hodgkin Lymphoma Study Group, although CHOP-21 remains the standard of care for older patients with PTCL, addition of etoposide to CHOP-21 or CHOP-14 significantly improved the event free survival (EFS) rate in younger patients with normal LDH levels, while the OS was not affected^11^. However, the benefit of etoposide on EFS was mainly confirmed in patients with ALK positive ALCL patients, and only a trend of benefit was found in patients with other PTCL entities (such as ALK negative ALCL, AITL, and PTCL-NOS)^11^. It is difficult to compare our efficacy data to standard CHOEP, given the lack of prospective data with CHOEP in patients with the same PTCL entities. However, compared with retrospective data reported by Liu et al.^12^, in which patients with PTCL achieved an ORR of 76.1%, CR of 56.5%, 3-year PFS of 29.9%, and OS of 47% when treated with the CHOEP regimen, our study did not show additional benefits when chidamide was added in the first-line setting. Although chidamide was shown to be highly active in relapsed/refractory AITL (50% ORR and 40% CR)^8^, we did not observe any benefits from chidamide in patients with untreated AITL.

To date, attempts to integrate HDAC inhibitors with upfront chemotherapy have mostly been early phase studies. Romidepsin was combined with CHOP in a phase 1b/2 study in 35 untreated PTCL patients, and the ORR was 68% with an OS of 76.5% and PFS of 57% at 18 months^13^. A phase 3 randomized controlled trial evaluating the efficacy and safety of romidepsin plus CHOP *vs.* CHOP in patients with untreated PTCL is ongoing (ClinicalTrials.gov Identifier: NCT01796002). Belinostat was combined with CHOP in a phase I study in untreated PTCL patients. Eighteen of 23 patients (78%) completed all 6 cycles of planned treatment, with 87% completed at least 4 cycles. The ORR was 89% (16/18), with a CR of 72%^14^. These results appeared to be superior to the results reported in the present study; however, the baseline characteristics of these studies were different. Approximately 30% of patients were elderly and 80% of patients had advanced disease in the current study.

In addition to supplementing chemotherapies, HDAC inhibitors can be combined with other novel agents. An epigenetic drug combination of HDAC and DNMT inhibitors target 2 epigenetic aberrations important in T-cell lymphomagenesis. A phase 1/2a trial evaluating azacitidine plus romidepsin for relapsed refractory lymphoma was conducted and showed a synergistic effect^15^. Furthermore, the combination of pralatrexate and romidepsin has been shown to be effective and safe for PTCLs^16^. Based on the limited data from these studies, the combination of novel agents may represent a promising strategy in the treatment of PTCL.

The percentage of ASCT was very low (7.1%) in our study, and a robust conclusion could not be reached concerning the benefit of ASCT; however, the 5-year PFS was reported to be 44% in patients who were consolidated with ASCT in the NLG-T-01 study^4^. Thus, it is currently recommended to conduct upfront consolidation with ASCT for eligible patients. Although brentuximab vedotin (BV) with cyclophosphamide, doxorubicin, and prednisone has shown clear benefits over CHOP in patients with untreated CD30-positive PTCL, subgroup analysis revealed no advantages of adding BV in patients with AITL and PTCL-NOS^17^. Comprehensive genomic analysis of PTCL is urgently needed to guide precision medicine.

The Chi-CHOEP regimen was generally well-tolerated and the toxicity profile was mainly characterized by expected hematological and gastrointestinal events. It has been demonstrated that romidepsin causes EBV reactivation by reversing the repressed state of genes crucial for EBV reactivation through histone acetylation in extranodal NK/T-cell lymphoma^18^. Our study did not show EBV reactivation throughout the duration of treatment with the Chi-CHOEP regimen, although the number of patients with positive EBV-DNA at baseline was too small to draw a robust conclusion. However, no HBV reactivation occurred in the present study.

## Conclusions

This large cohort phase 1b/2 study showed that Chi-CHOEP was well-tolerated with modest efficacy in previously untreated PTCL patients. Compared with previously reported data, Chi-CHOEP did not reveal clear benefits from adding chidamide, a novel HDAC inhibitor, to the CHOEP regimen in patients with untreated PTCL, which needs to be validated in phase 3 randomized controlled trials.
